# Supplementary material for: Manipulation of sterol homeostasis for the production of 24-epi-ergosterol in industrial yeast
Source: Nat Commun. 2023 Jan 27;14:437. doi: 10.1038/s41467-023-36007-z (PMC9883489; doi:10.1038/s41467-023-36007-z)
Supplement: Supplementary file 1 — Supplementary Information [file 41467_2023_36007_MOESM1_ESM.pdf]

Manipulation of sterol homeostasis for the production of 24-epi-ergosterol in industrial yeast

Jiang *et al.*

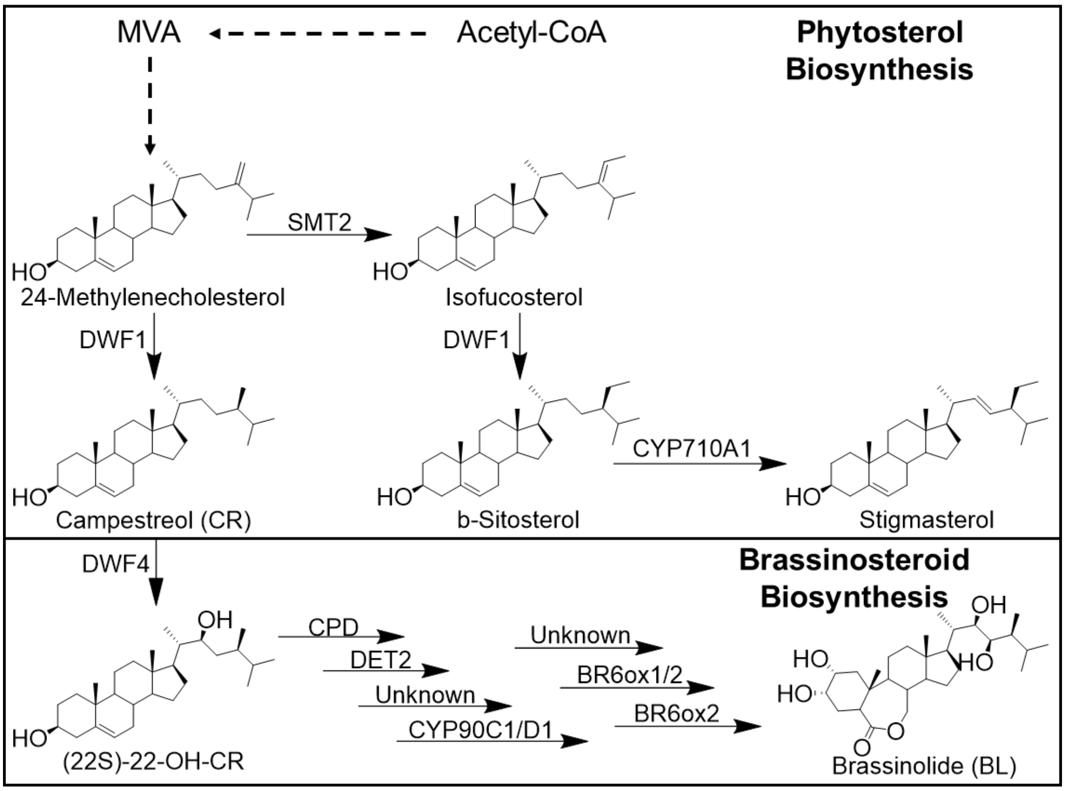

Supplementary Fig. 1. A simplified BL biosynthetic pathway from acetyl-CoA. In plants, 24-methylenecholesterol and isofucosterol are catalyzed by DWF1 to synthesize campesterol and β-stiosterol, respectively. BL is proposed to be biologically synthesized from campesterol with 8 enzymatic reactions[1]. Unfortunately, several of the BL biosynthetic pathway enzymes have not been identified yet.

```
AtDWF1 MSDLQTPLVRPKRKKTWVDYFVKFRWIIVIFIVLPFSATFYFLIYLGDMWSESKSFEKRQ 60
BrDWF1 MSDLQAPLVRPKRKKTWVDYFVKFRWIIVIFVVLPISATLYFLIYLGDMWSESKSYEKRR 60
ArDWF1 MSDLEVP-LRPKRKKIWVDYFVQFRWIIVIFLVLPISFTLYFLTYLGDVRSESKSFKQRQ 59
CsDWF1 MSDLEAP-LRPKRKKIWVDYFVQFRWIIVIFIVLPISCTLYFLTYLGDVKSERKSYKQRQ 59

AtDWF1 KEHDENVKKVIKRLKGRDASKDGLVCTARKPWIAVGMRNVDYKRARHFEVDLGEFRNILE 120
BrDWF1 KEHDQNVAKVIKRLKERDAAKDGLVCTARKPWIAVGMRNVDYKRARHFEVDLGEFRNILE 120
ArDWF1 REHDENVEKVVKRLKERNPKKDGLVCTARKPWISVGMRNVDYKRARHFEVDLSAFRNVLD 119
CsDWF1 KEHDENVQKVVKRLKQRNPSKDGLVCTARKPWIAVGMRNVDYKRARHFEVDLSAFRNVLE 119

AtDWF1 INKEKMTARVEPLVNMGQISRATVPMNLSLAVVAELDDLTVGGLINGYGIEGSSHIYGLF 180
BrDWF1 INKEKMIARVEPLVNMGQISRATVPMNLSLAVVAELDDLTVGGLINGYGIEGSSHLYGLF 180
ArDWF1 IDKERMIARVEPLVNMGQISRVTVPMNLSLAVVAELDDLTVGGLINGYGIEGSSHLYGLF 179
CsDWF1 IDRDRMIAKVEPLVNMGQISRVTVPMNLSLAVVAELDDLTVGGLINGYGIEGSSHIYGLF 179

AtDWF1 ADTVEAYEIVLAGGELVRATRDNEYSDLYYAIPWSQGTLGLLVAAEIRLIKVKEYMRLTY 240
BrDWF1 ADTVVAYEIVLAGGELVRATKDNEYSDLFYAIPWSQGTLGLLVAAEIKLIPVKEYMKLTY 240
ArDWF1 SDTVVSYEIVLADGRLVRATKDNQYSDLFYAIPWSQGTLGLLVAAEVKLIPVKEYMKVTY 239
CsDWF1 SDTVVAYEIVLADGSVVRATKDNEYSDLFYAIPWSQGTLGLLVSAEIKLIPIKEYMKLTY 239

AtDWF1 IPVKGDLQALAQGYIDSFAPKDGDK---SKIPDFVEGMVYNPTEGVMMVGTYASKEEAKK 297
BrDWF1 IPVKGDLQTLAQGYMDSFAPKDGDT---SKIPDFVEGMVYNPTEGVMMVGTYASKEEAKK 297
ArDWF1 KPIVGNLKELAQGYIDSFAPRDGDQDNPEKVPDFVETMIYSPTEGVCMTGRYASKEEAKK 299
CsDWF1 KPVIGNIQDLAQGYVDSFAPRDGDQDNPDKVPDFVETMIYSPTEGVCMTGVYASKEEAKK 299

AtDWF1 KGNKINNVGWWFKPWFYQHAQTALKKGQFVEYIPTREYYHRHTRCLYWEGKLILPFGDQF 357
BrDWF1 KGNKINNVGWWFKPWFYQHAQTALKKGEFVEYIPTREYYHRHTRCLYWEGKLILPFGDQF 357
ArDWF1 KGNKINSVGWWFKPWFYQHAQTALKKGEFVEYIPTRDYYHRHTRSLYWEGELILPFADQW 359
CsDWF1 KGNVINNVGWWFKPWFYQHAQKALKKGEFVEYIPTREYYHRHTRCLYWEGKLILPFGDQW 359

AtDWF1 WFRYLLGWLMPPKVSLLKATQGEAIRNYYHDMHVIQDMLVPLYKVGDALEWVHREMEVYP 417
BrDWF1 WFRFLFGWLMPPKVSLLKATQGEAIRNYYHDMHVIQDMLVPLYKVGDALEWVHREMEVYP 417
ArDWF1 WFRFLLGWLMPPKVSLLKATQGEAIRNYYHEMHVIQDMLVPLYKVGDALEFVHREMELYP 419
CsDWF1 WFRFLFGWLMPPKVSLLKATQGEAIRNYYHEMHVIQDMLVPLYKVGDALEWVDREMEVYP 419

AtDWF1 IWLCPHKLFKQPIKGQIYPEPGFEYENRQGDTEDAQMYTDVGVYYAPGCVLRGEEFDGSE 477
BrDWF1 IWLCPHKLYKAPIKQQIYPEPGFEYERRQGDTEDAQMYTDVGVYYAPGPVLRGEEFDGSE 477
ArDWF1 VWLCPHRLFKLPVKPMISPEPGFELQRRQGDTHYAQMYTDILLCYAPGPVLRGEQFDGAE 479
CsDWF1 IWLCPHRLYKLPMKTMVYPEPGFELHRRQGDTQYAQMYTDVGVYYSPGPVLRGEVFDGIE 479

AtDWF1 AVRRMEKWLIENHGFQPQYAVSELDEKSFWRMFNGELYEECRKKYRAIGTFMSVYYKSKK 537
BrDWF1 AVRKMEKWLIENGGFQPQYAVSELDEKSFWRMFDGDLYEHCRKKYRAVGTFMSVYYKSKK 537
ArDWF1 AVHRMENWLIENHGYEPQYTVSELSEKNFWRMFDGGLYEQCRRKYGAVGTFMSVYYKSKK 539
CsDWF1 AVRRMEKWLIENHGFQPQYAVSELDEKNFWRMFDAGLYEHCRRKYGAVGTFMSVYYKSKK 539

AtDWF1 GRKTEKEVREAEQAHLETAYAEAD 561
BrDWF1 GRKTEKEVREAEQAHLETAYAEAD 561
ArDWF1 GRKTEKEVQEAEQAILESPDAEVA 563
CsDWF1 GRKTEKEVQEAEQAQLDTAYAEVD 563
```

Supplementary Fig. 2. Alignment of the amino acid sequences of DWF1 orthologues. *DWF1* genes from *Arabidopsis thaliana (AtDWF1)*(Q39085.2)[2], *Ajuga reptans (ArDWF1)*(BAS68578.1)[3], *Brassica rapa (BrDWF1)*(VDC72099.1), and *Cannabis sativa (CsDWF1)*(XP_030508560.1) were codon-optimized for yeast expression and chemically synthesized. The sequences of BrDWF1 and CsDWF1 were obtained by PSI-BLAST based on AtDWF1 and ArDWF1.

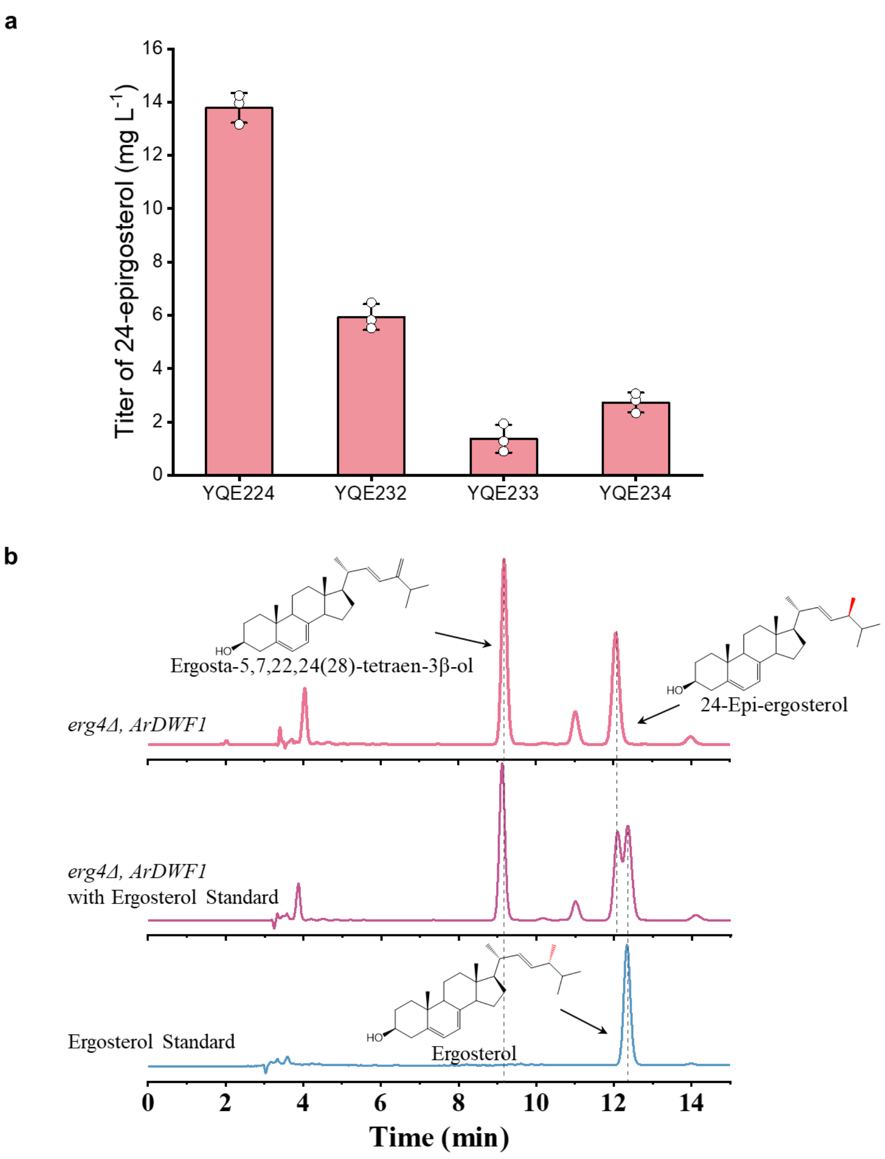

Supplementary Fig. 3. *De novo* biosynthesis of 24-epi-ergosterol in engineered yeast strains. a The expression cassettes of *ArDWF1* (YQE224), *AtDWF1* (YQE232), *BrDWF1* (YQE233), and *CsDWF1* (YQE234) were integrated into the genome of YQE102 to evaluate their performance for 24-epi-ergosterol production. **b** HPLC analysis for ergosterol standard, *erg4Δ-ArDWF1* strain with ergosterol standard and *erg4Δ-ArDWF1* strain respectively. **a** Data are presented as mean values ± SD from three independent biological replicates (n=3), the circles represent individual data points. Source data are provided as a Source Data file**.**

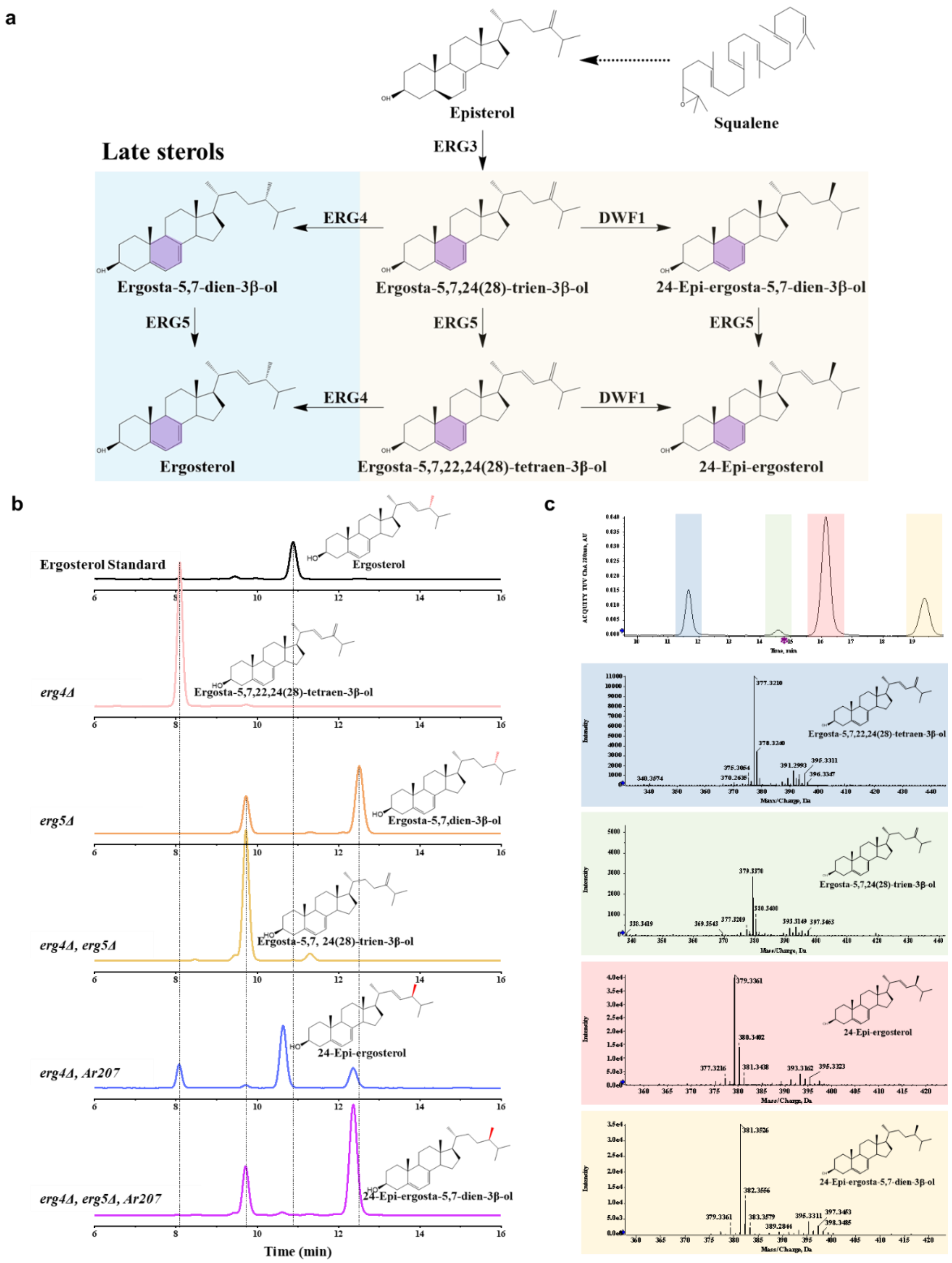

Supplementary Fig. 4. LCMS analysis of 24-epi-ergosterol and its biosynthetic precursors. a Late sterol biosynthetic pathway including ERG4, ERG5, and DWF1. **b** HPLC profiles for ergosterol standard, *erg4Δ* strain (producing ergosta-5,7,22,24(28)-tetraen-3β-ol), *erg5Δ* strain (producing ergosta-5,7-dien-3β-ol), *erg4Δ-erg5Δ* strain (producing ergosta-5,7,24(28)-trien-3β-ol), *erg4Δ-Ar207* strain (producing 24-epi-ergosterol), and *erg4Δ-erg5Δ-Ar207* strain (producing 24-epi-ergosta-5,7-dien-3β-ol). **c** MS verification of the HPLC peaks as ergosta-5,7,22,24(28)-tetraen-3β-ol, ergosta-5,7,24(28)-trien-3β-ol, 24-epi-ergosterol, and 24-epi-ergosta-5,7-dien-3β-ol, respectively. Source data are provided as a Source Data file.

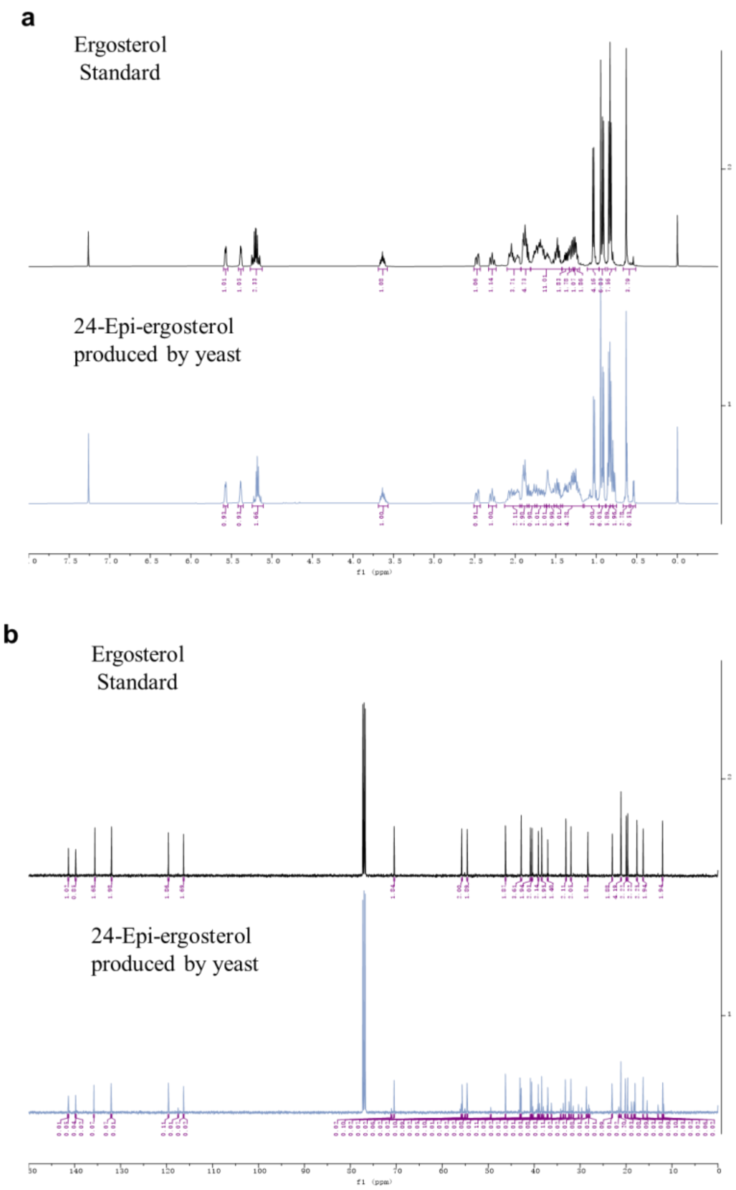

Supplementary Fig. 5. NMR profiles of ergosterol standard and 24-epi-ergosterol. **a** ^{1}H NMR spectroscopy. 24-Epi-ergosterol (500 MHz, $CDCl_3$) δ 5.57 (dd, 1H), 5.38 (dt, 1H), 5.24-5.11 (m, 2H), 3.63 (tq, 1H), 2.47 (ddd, 1H), 2.28 (ddd, 1H), 2.13-1.92 (m, 2H), 1.88 (dddd, 3H), 1.85-1.73 (m, 1H), 1.76-1.62 (m, 1H), 1.61 (dd, 1H), 1.61-1.49 (m, 1H), 1.52-1.42 (m, 1H), 1.44-1.17 (m, 4H), 1.15-0.97 (m, 3H), 0.96-0.89 (m, 6H), 0.88-0.80 (m, 6H), 0.79 (td, 2H), 0.63 (d, 3H), 0.54 (d, 1H). **b** ^{13}C NMR spectroscopy. 24-Epi-ergosterol (126 MHz, $CDCl_3$) δ 141.47, 141.37, 139.80, 139.77, 135.82, 132.07, 131.98, 119.60, 117.48, 116.30, 116.27, 71.08, 70.47, 55.86, 55.68, 55.06, 54.59, 54.52, 49.46, 46.25, 43.09, 42.94, 42.83, 40.81, 40.62, 40.56, 40.27, 39.20, 39.10, 38.85, 38.39, 38.00, 37.15, 37.04, 36.26, 34.22, 33.66, 33.23, 32.43, 32.01, 31.49, 30.35, 29.66, 28.61, 28.43, 28.11, 27.97, 23.06, 23.04, 21.56, 21.13, 20.22, 20.18, 19.66, 18.84, 18.28, 18.05, 16.30, 15.40, 13.06, 12.06, 12.02, 11.84. Source data are provided as a Source Data file.

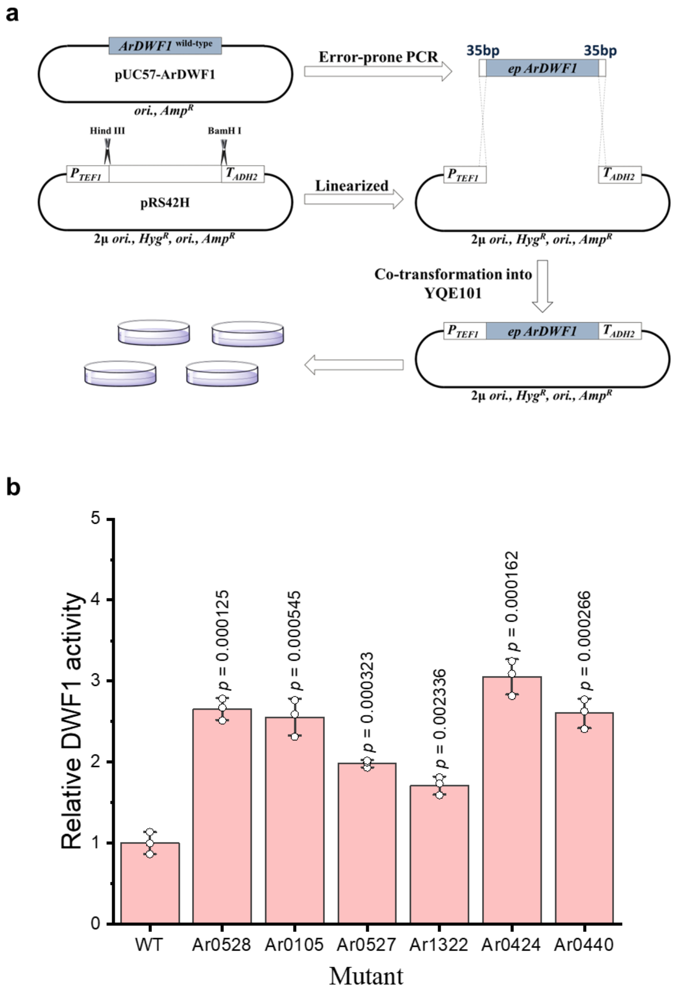

Supplementary Fig. 6. Directed evolution of ArDWF1 for improved production of 24-epi-ergosterol in engineered yeast strains. a The procedure of library construction using homologous recombination in yeast. pRS42H was linearized by *Hin*dIII and *Bam*HI. The mutant library, with 35 bp homologous arms to pRS42H was generated by error-prone PCR. Co-transformation of mutant fragment and linearized plasmid into YQE101 results in recombination and library construction. **b** Positive mutants obtained from directed evolution. Six mutants with a total of 10 mutations were obtained in the first round of directed evolution. The DWF1 activity was defined as the ratio of HPLC peak areas of 24-epi-ergosterol to ergosta-5,7,22,24(28)-tetraen-3β-ol. **b** Data are presented as mean values ± SD from three independent biological replicates (n=3), the circles represent individual data points. Significance (*p*-value) was evaluated by two-sided *t*-test, no significance (n.s.) presents $p > 0.05$. Source data are provided as a Source Data file**.**

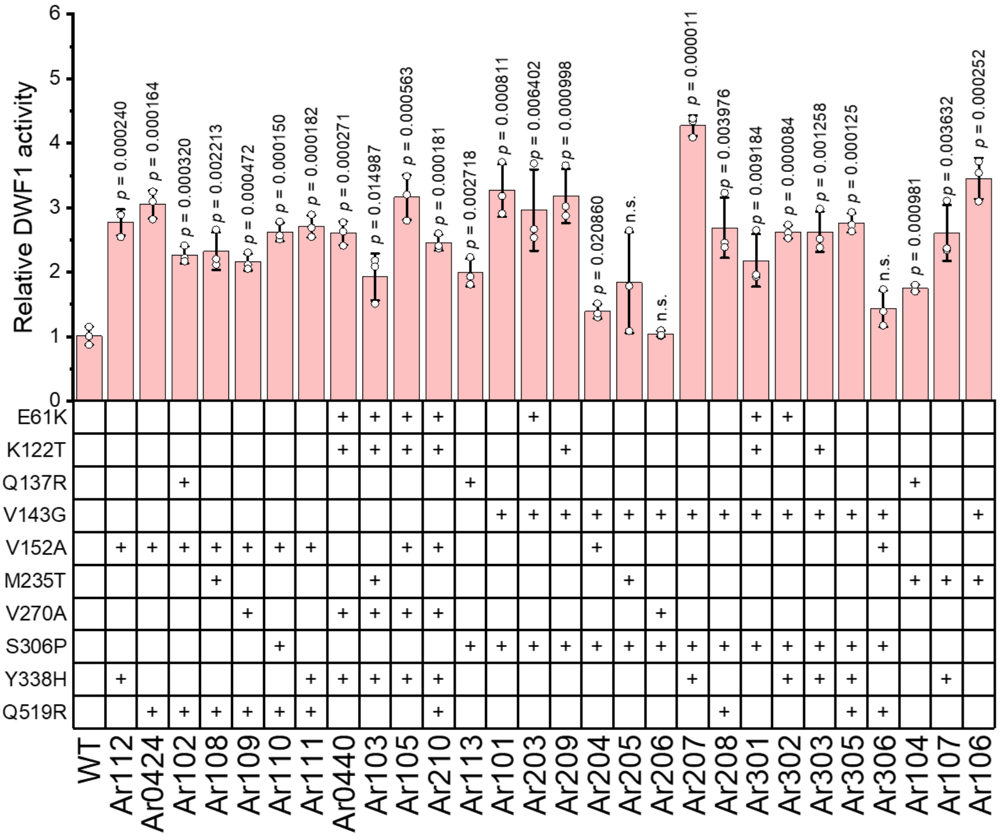

Supplementary Fig. 7. The activity of DWF1 combinatorial mutants. The second round of directed evolution via DNA shuffling resulted in the construction of a series of combinatorial mutants, whose activity was evaluated by episomal plasmid transformation into YQE101 (for relative DWF1 activity). Data are presented as mean values ± SD from three independent biological replicates (n=3), the circles represent individual data points. Significance (*p*-value) was evaluated by two-sided *t*-test, no significance (n.s.) presents $p > 0.05$. Source data are provided as a Source Data file**.**

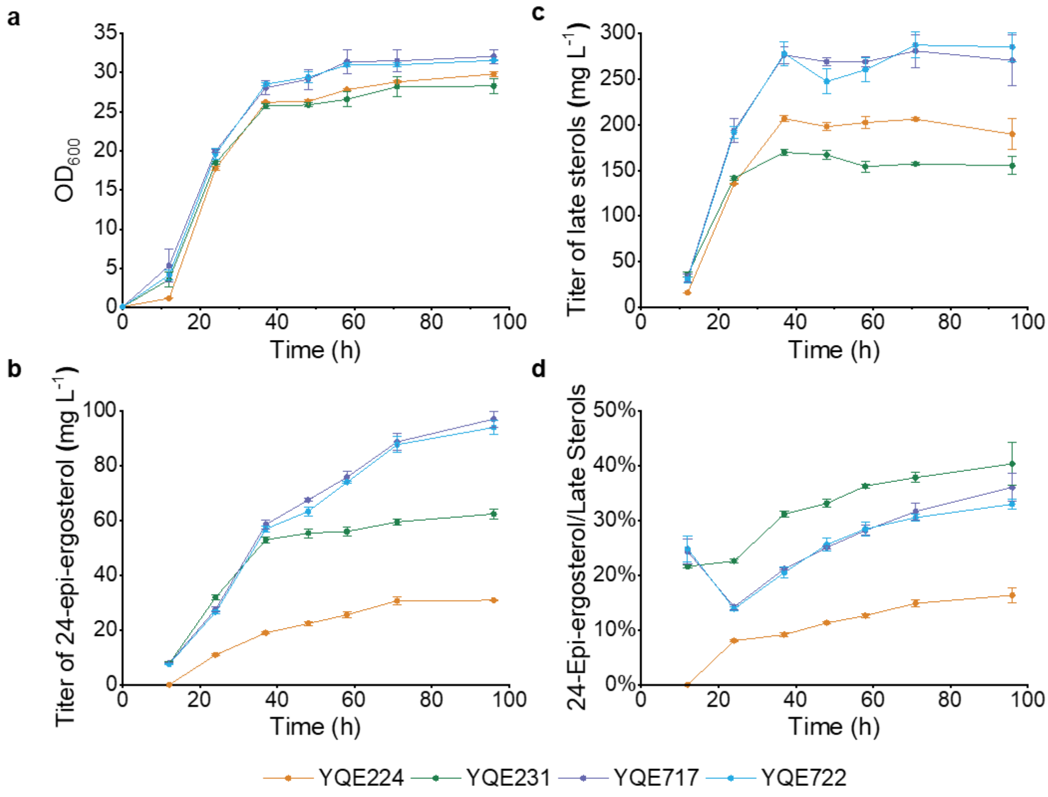

Supplementary Fig. 8. Fermentation profiles of YQE224, YQE231, YQE717, and YQE722 in shake flasks. Fermentation of YQE224, YQE231, YQE717, and YQE722 was performed in 250 mL shake flasks with 50 mL YPD medium containing 20 g/L glucose for 96 h, to obtain the time courses of cell growth **a**, titer of 24-epi-ergosterol **b**, titer of late sterols **c**, and the ratio of 24-epi-ergosterol to late sterols **d**. Data are presented as mean values ± SD from three independent biological replicates (n=3). Source data are provided as a Source Data file**.**

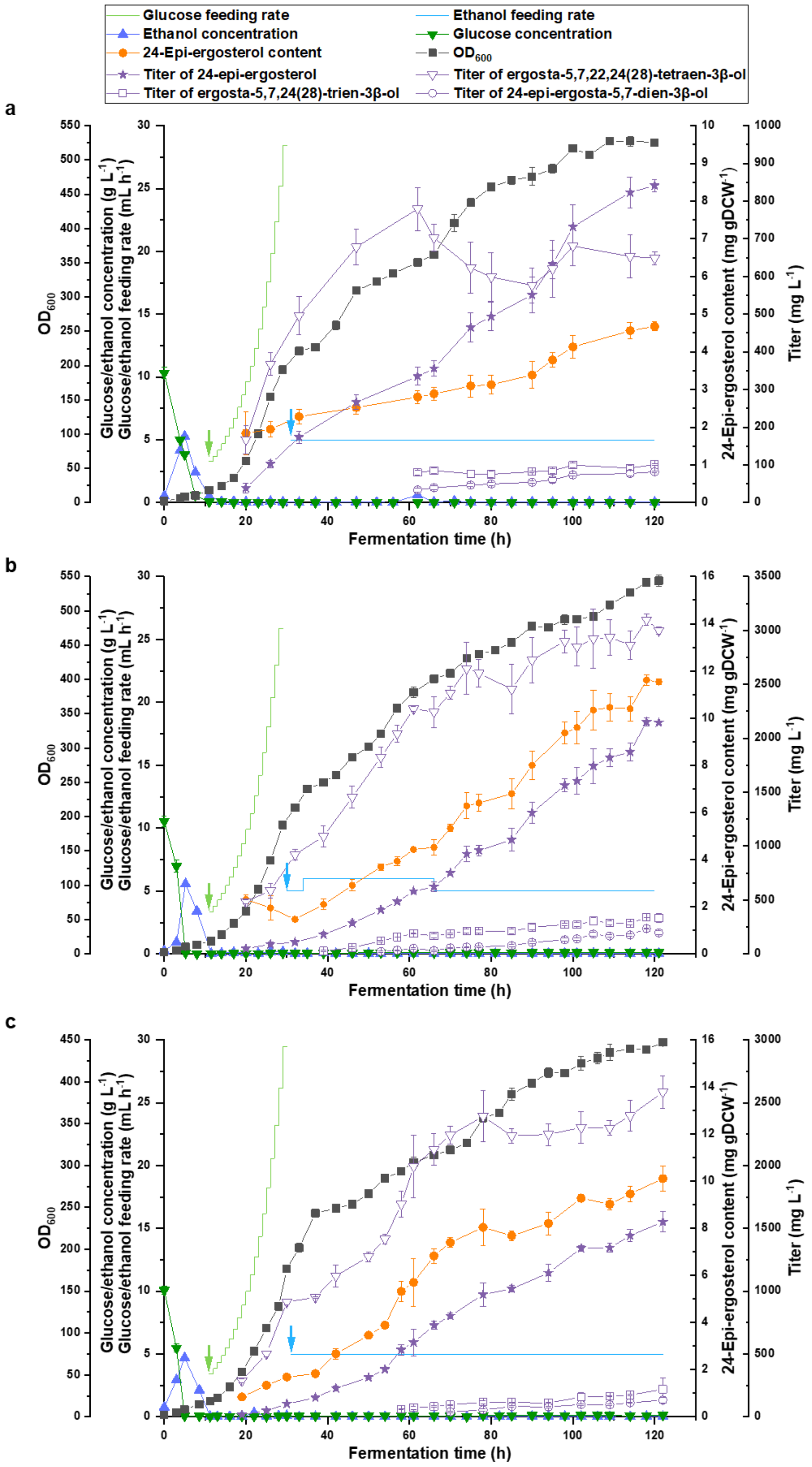

Supplementary Fig. 9. Fed-batch fermentation for YQE231, YQE717, and YQE722. a YQE231. **b** YQE717. **c** YQE722. The green and blue arrows indicated the start of glucose and ethanol feeding, respectively. Data are presented as mean values ± SD from three independent biological replicates (n=3). Source data are provided as a Source Data file.

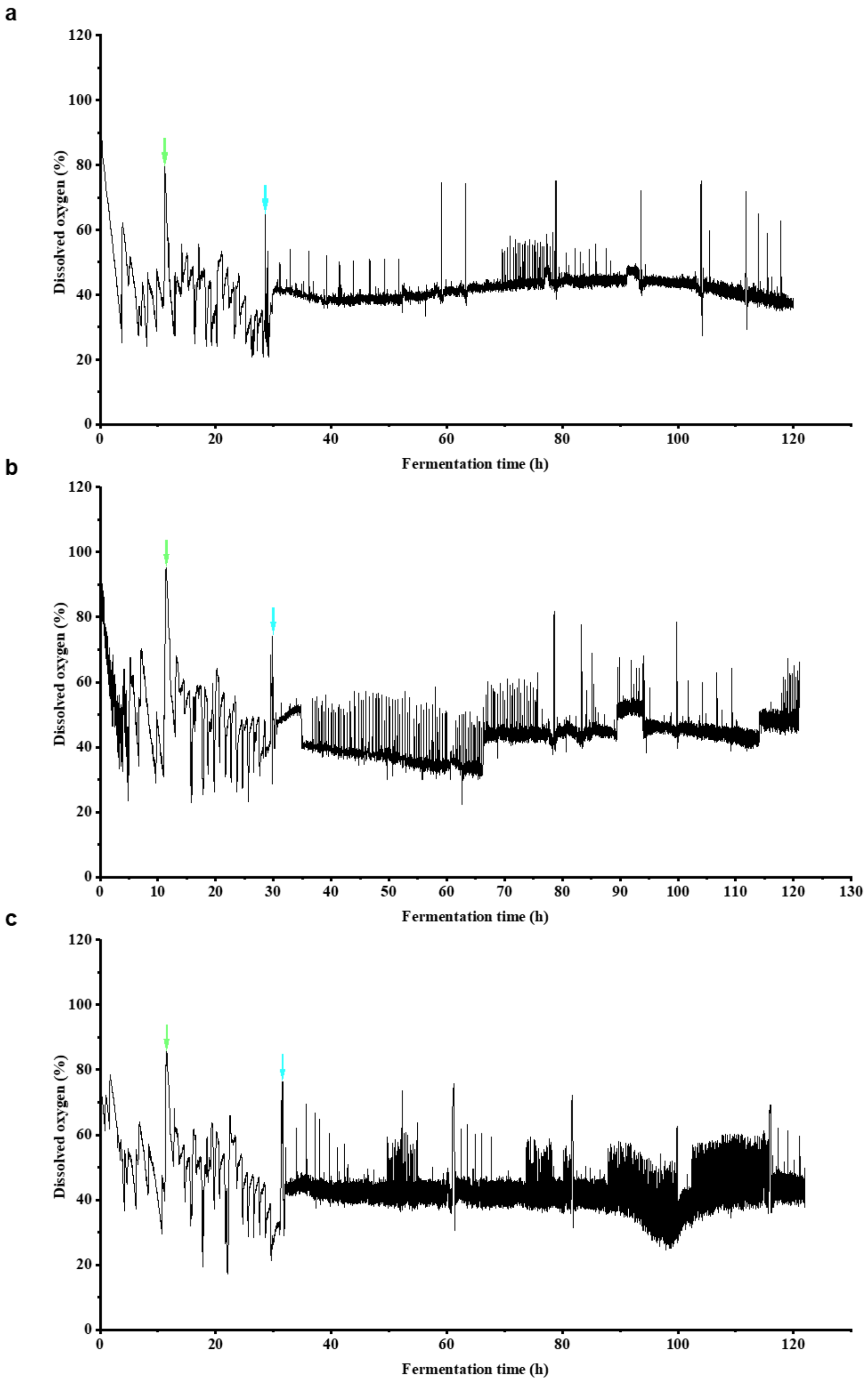

Supplementary Fig. 10. Dissolved oxygen curves of fed-batch fermentation for YQE231, YQE717, and YQE722. **a** YQE231. **b** YQE717. **c** YQE722. The green and blue arrows indicated the start of glucose and ethanol feeding, respectively. Source data are provided as a Source Data file.

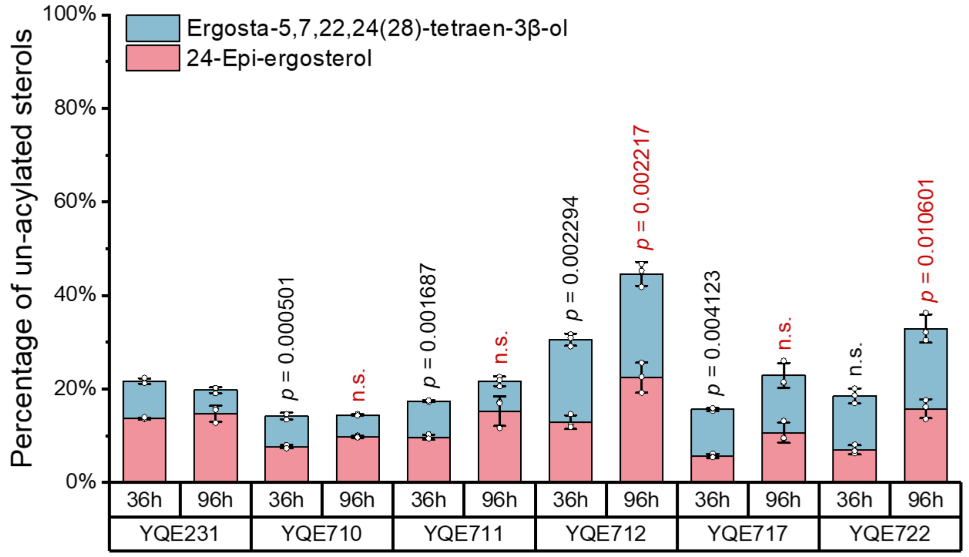

Supplementary Fig. 11. The percentage of un-acylated sterols in engineered yeast strains. The un-acylated form of 24-epi-ergosterol and ergosta-5,7,22,24(28)-tetraen-3β-ol were quantified by HPLC. Data are presented as mean values ± SD from three independent biological replicates (n=3), the circles represent individual data points. The statistical evaluation (*p*-value) of the percentage of un-acylated sterols in 36 h (black) and 96 h (red star) were performed by two-sided *t*-test separately, no significance (n.s.) presents $p > 0.05$. Source data are provided as a Source Data file**.**

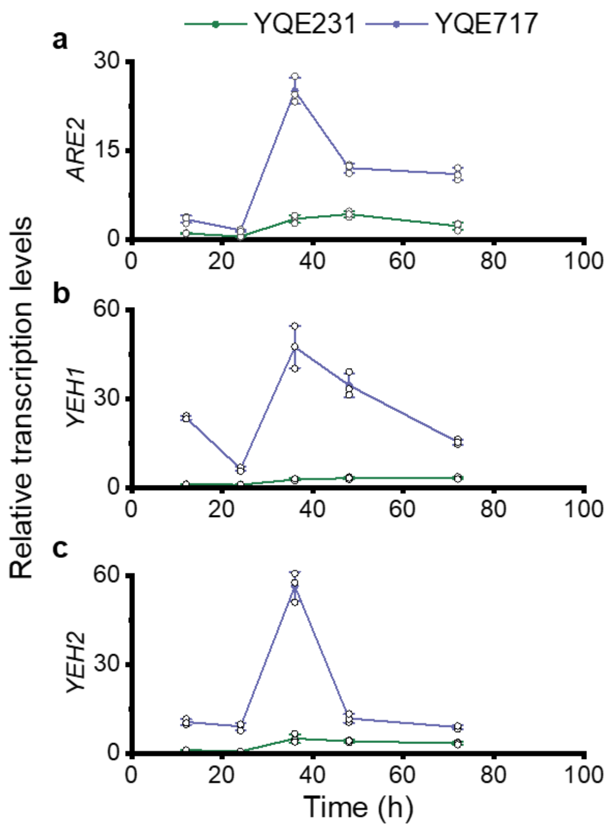

Supplementary Fig. 12. Relative transcription level of *ARE2*, *YEH1*, *YEH2* in YQE717 and YQE231. a ARE2. **b** YEH1. **c** YEH2. Samples collected at 12 h, 24 h, 36 h, 48 h, and 72 h during fermentation of the two strains in shake flasks were subject to qPCR analysis. The transcription level of YQE231 for the three genes at 12 h was set to 1. Data are presented as mean values ± SD from three independent biological replicates (n=3). Source data are provided as a Source Data file**.**

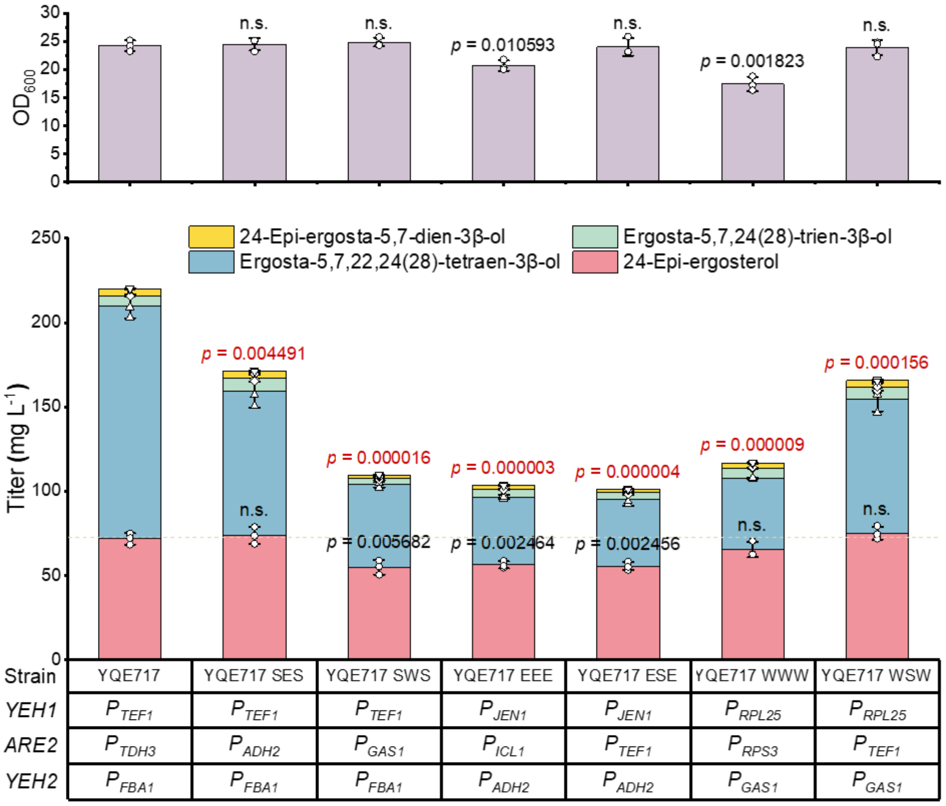

Supplementary Fig. 13. Quantification of late sterols with different promoter combinations for the expression of *ARE2*, *YEH1*, and *YEH2* in YQE231. Details on the properties of the selected promoters were provided in Supplementary Table 3. Data are presented as mean values ± SD from three independent biological replicates (n=3), the circles represent individual data points. Significance (*p*-value) of the titer of total late sterols (red) and 24-epi-ergosterol (black) were evaluated by two-sided *t*-test, no significance (n.s.) presents $p > 0.05$. Source data are provided as a Source Data file**.**

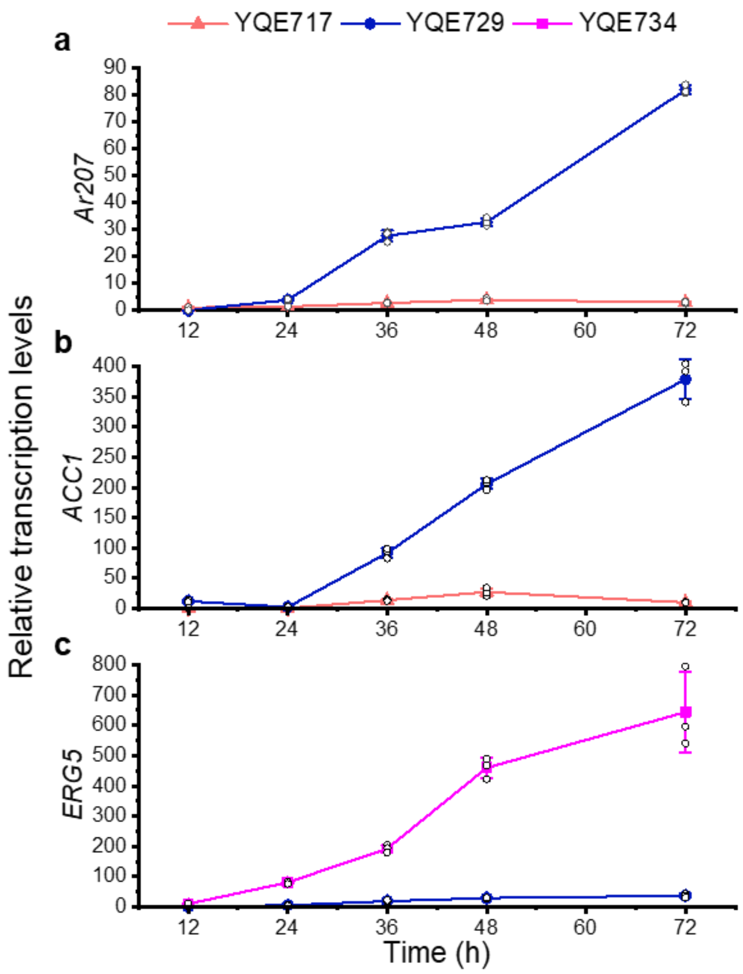

Supplementary Fig. 14. Relative transcription level of *Ar207*, *ACC1*, and *ERG5* in YQE717, YQE729, and YQE734. a *Ar207*. **b** *ACC1*. **c** *ERG5*. Samples collected at 12 h, 24 h, 36 h, 48 h, and 72 h during the fermentation of these strains in shake flasks were used for qPCR analysis to determine the transcription level. The transcription level of YQE717 for *Ar207* and *ACC1* as well as YQE729 for *ERG5* at 12 h were set to 1. Data are presented as mean values ± SD from three independent biological replicates (n=3). Source data are provided as a Source Data file**.**

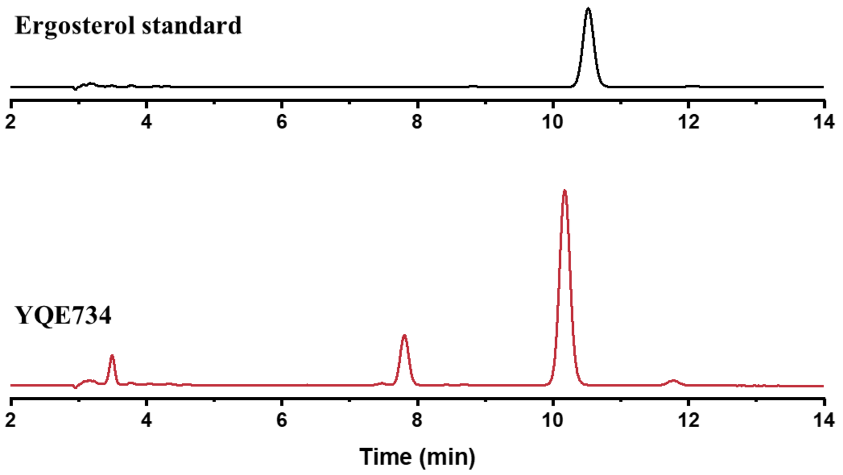

Supplementary Fig. 15. HPLC profiles for ergosterol standard and YQE734. Source data are provided as a Source Data file.

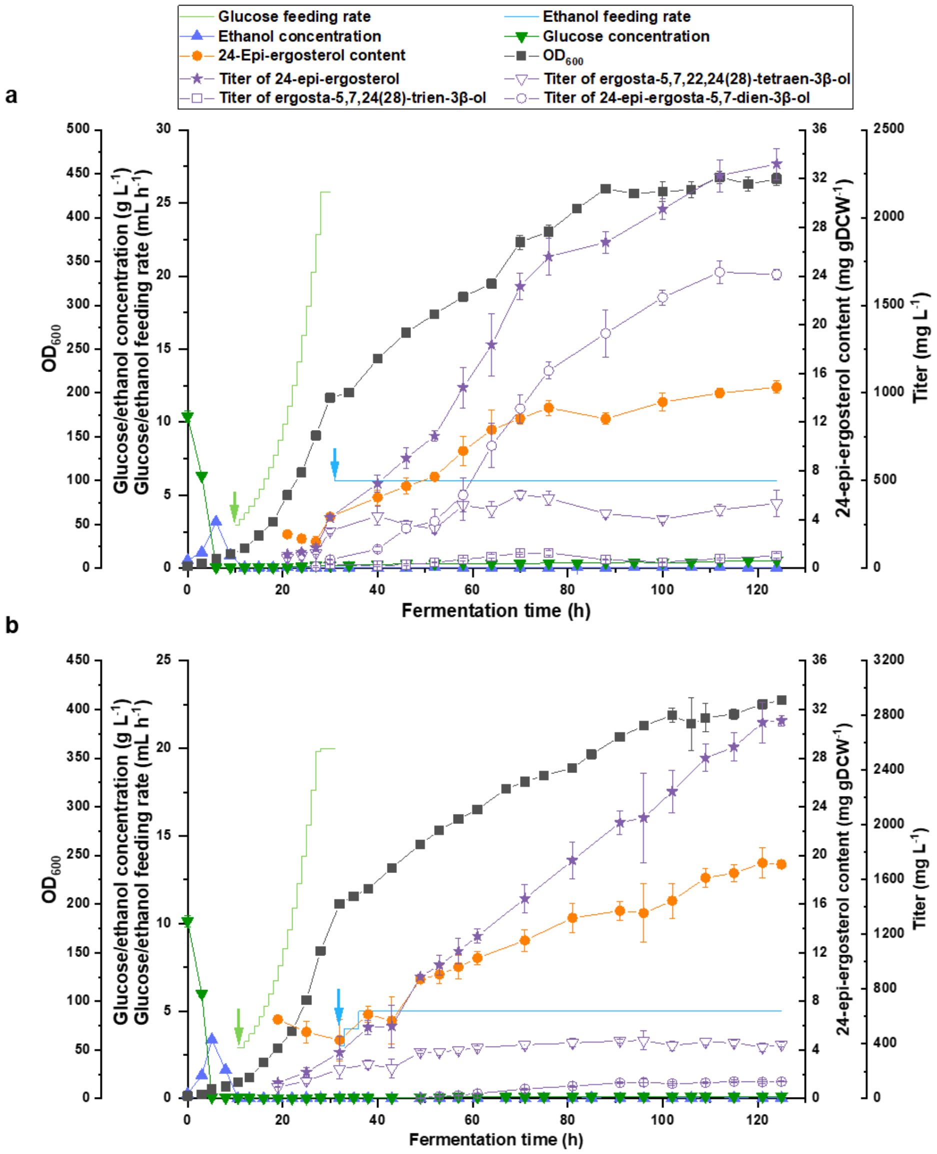

Supplementary Fig. 16. Fed-batch fermentation for YQE729 and YQE734. a YQE729. **b** YQE734. The green and blue arrows indicated the start of glucose and ethanol feeding, respectively. Data are presented as mean values ± SD from three independent biological replicates (n=3). Source data are provided as a Source Data file.

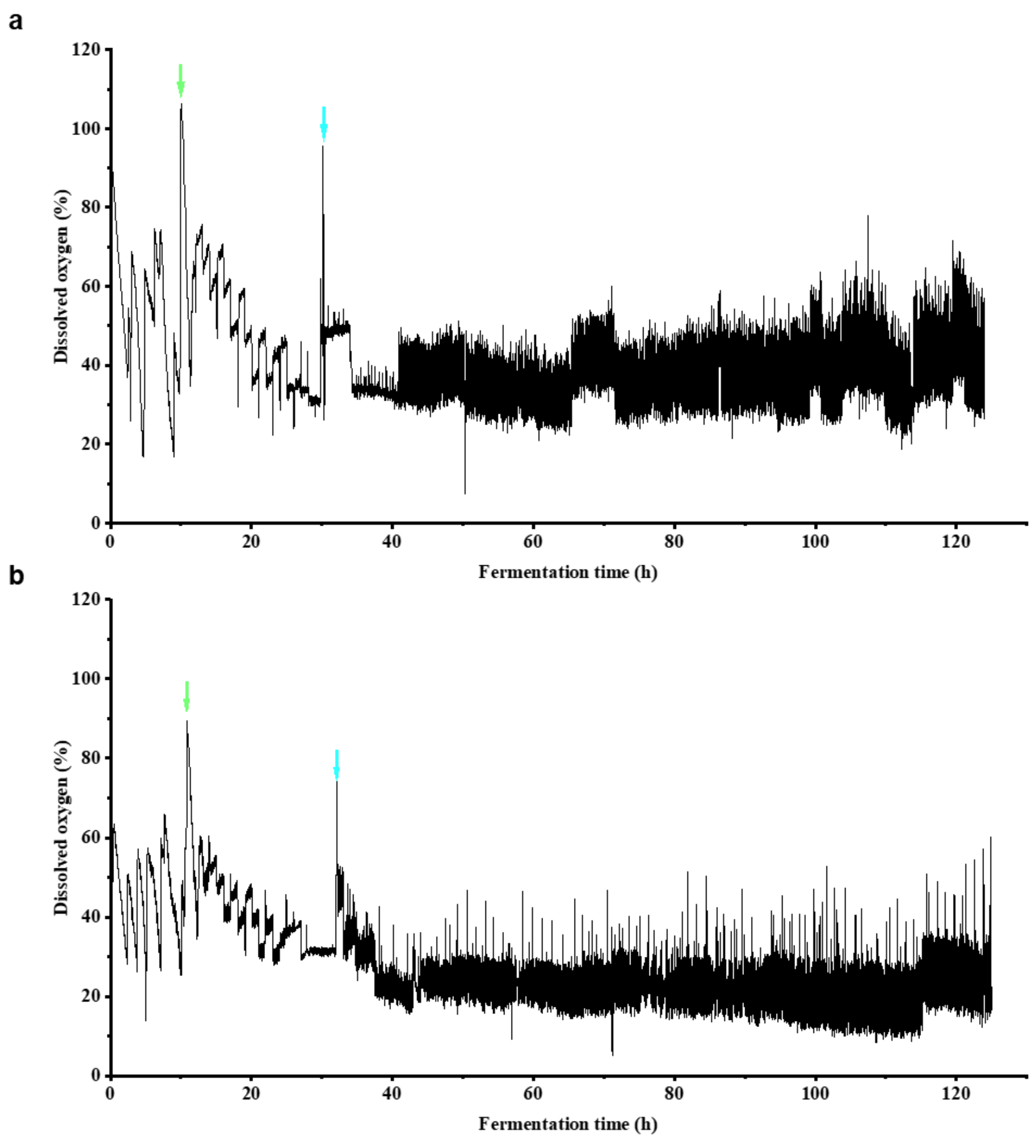

Supplementary Fig. 17. Dissolved oxygen curves of fed-batch fermentation for YQE729 and YQE734. a YQE729. **b** YQE734. The green and blue arrows indicated the start of glucose and ethanol feeding, respectively. Source data are provided as a Source Data file**.**

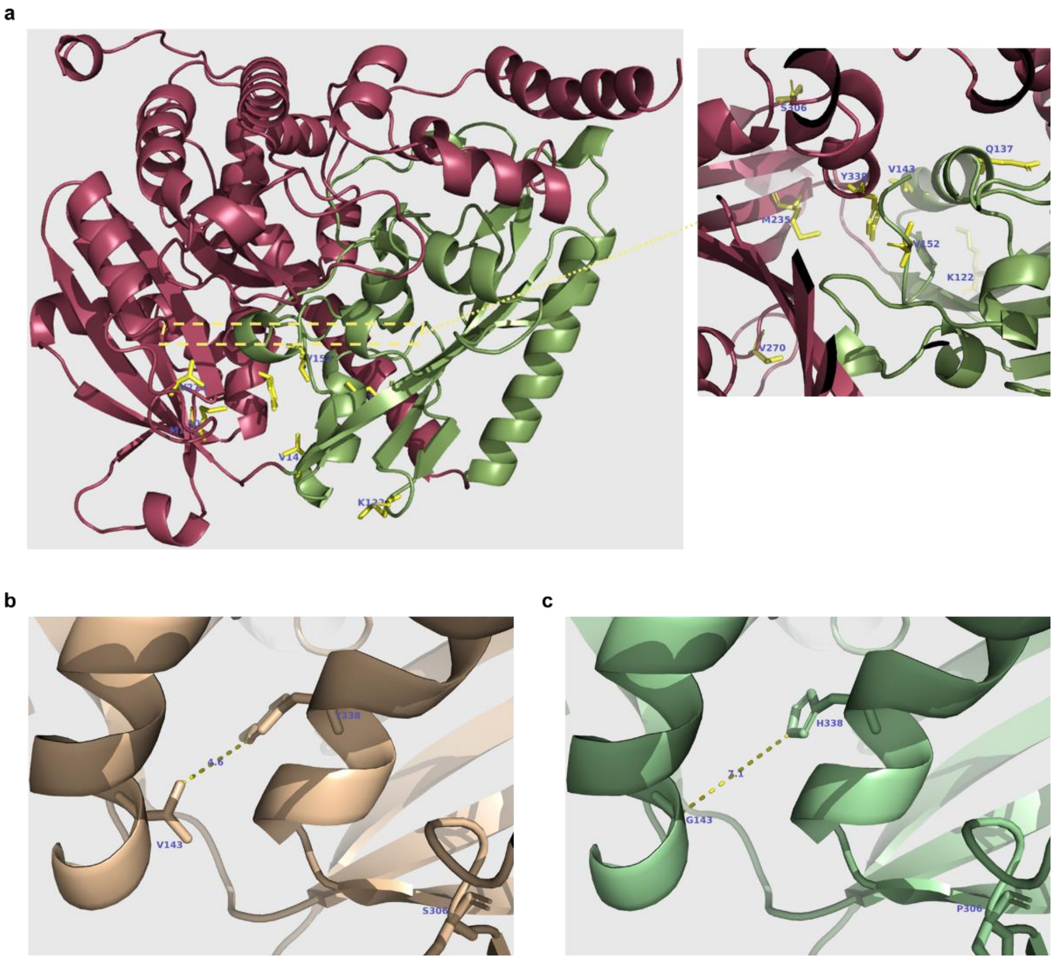

Supplementary Fig. 18. Molecular mechanisms for improved activity of the evolved ArDWF1 mutants. a Predicted structure of ArDWF1 by AlphaFold2 (https://colab.research.google.com/github/sokrypton/ColabFold/blob/main/AlphaFold2.ipynb#scrollTo=kOblAo-xetgx). FAD-binding domain and positive sites (K122, Q137, V143, V152, M235, V270, S306, and Y338) were highlighted in green and yellow, respectively. The residues at 143, 338, and 306 in ArDWF1 **b** and Ar207 **c** were compared.

Supplementary Table 1. Activity comparison among BL, EBL, and HBL.

Species	Bioassay	BL	EBL	HBL	Reference
Bean	1st internode test	100	36	33	4
	2nd internode test	100	10	0.1	
Radish	Elongation test	100	10	10	5
Tomato		100	10	1	
Rice (Arborio)	Rice-lamina inclination test	100	10	100	6
Rice (Bahia)		100	5	5	7
Rice (Tan-[illegible]		100	10	25	8
Tomato	Cell-wall-bound Invertase activity	100 (0.2 μM)	79.6 (2 μM)	67.6 (2 μM)	9
	Sucrose uptake	100 (0.2 μM)	73.5 (2 μM)	71.4 (2 μM)	
Barberry (Maria)	Total soluble	100	96.9		10
	Free amino acids	100	77.6		
Barberry (Red Rocket)	Total soluble	100	65.4		
	Free amino acids	100	96.8		

Supplementary Table 2. Mutants obtained from the first round of directed evolution.

Mutants	Mutation sites			
Ar0105	V143G			
Ar0424	V152A	Q519R		
Ar0440	E61K	K122T	V270A	Y338H
Ar0527	S306P			
Ar0528	M235T			
Ar1322	Q137R			

Supplementary Table 3. Yeast promoter activity values under different growth conditions. The data were adapted from Keren *et al.*[11].

Strain	Carbon source					
	Glucose			Ethanol		
YQE717	P_{TEF1}	P_{TDH3}	P_{FBA1}	P_{TEF1}	P_{TDH3}	P_{FBA1}
	2.159565	3.234124	1.911226	0.430327	0.510003	0.212029
YQE717 SES	P_{TEF1}	P_{ADH2}	P_{FBA1}	P_{TEF1}	P_{ADH2}	P_{FBA1}
	2.159565	0.028053	1.911226	0.430327	0.539263	0.212029
YQE717 SWS	P_{TEF1}	P_{GAS1}	P_{FBA1}	P_{TEF1}	P_{GAS1}	P_{FBA1}
	2.159565	1.069321	1.911226	0.430327	0.205553	0.212029
YQE717 EEE	P_{JEN1}	P_{ICL1}	P_{ADH2}	P_{JEN1}	P_{ICL1}	P_{ADH2}
	0.012039	0.0115	0.028053	0.817225	0.706364	0.539263
YQE717 ESE	P_{JEN1}	P_{TEF1}	P_{ADH2}	P_{JEN1}	P_{TEF1}	P_{ADH2}
	0.012039	2.159565	0.028053	0.817225	0.430327	0.539263
YQE717 WWW	P_{RPL25}	P_{RPS3}	P_{GAS1}	P_{RPL25}	P_{RPS3}	P_{GAS1}
	1.077	1.067463	1.069321	0.178034	0.175343	0.205553
YQE717 WSW	P_{RPL25}	P_{TEF1}	P_{GAS1}	P_{RPL25}	P_{TEF1}	P_{GAS1}
	1.077	2.159565	1.069321	0.178034	0.430327	0.205553

Supplementary references

1 Wei, Z. Y. & Li, J. Brassinosteroids regulate root growth, development, and symbiosis. *Mol Plant* **9**, 86-100 (2016).

2 Takahashi, T., Gasch, A., Nishizawa, N. & Chua, N. H. The *DIMINUTO* gene of *Arabidopsis* is involved in regulating cell elongation *Gene Dev* **9**, 97-107 (1995).

3 Tsukagoshi, Y. *et al. Ajuga* Δ24-sterol reductase catalyzes the direct reductive conversion of 24-methylenecholesterol to campesterol. *J Biol Chem* **291**, 8189-8198 (2016).

4 Thompson, M. J. *et al.* Synthesis of brassinosteroids and relationship of structure to plant growth-promoting effects. *Steroids* **39**, 89-105 (1982).

5 Takatsuto, S. *et al.* Structure activity relationship of brassinosteroids. *Phytochemistry* **22**, 2437-2441 (1983).

6 Takatsuto, S., Yazawa, N., Ikekawa, N., Morishita, T. & Abe, H. Synthesis of (24R)-28-homobrassinolide analogs and structure activity relationships of brassinosteroids in the rice-lamina inclination test. *Phytochemistry* **22**, 1393-1397 (1983).

7 Brosa, C. Biological effects of brassinosteroids. *Crit Rev Biochem Mol* **34**, 339-358 (1999).

8 Fujioka, S., Noguchi, T., Takatsuto, S. & Yoshida, S. Activity of brassinosteroids in the dwarf rice lamina inclination bioassay. *Phytochemistry* **49**, 1841-1848 (1998).

9 Goetz, M., Godt, D. E. & Roitsch, T. Tissue-specific induction of the mRNA for an extracellular invertase isoenzyme of tomato by brassinosteroids suggests a role for steroid hormones in assimilate partitioning. *Plant J* **22**, 515-522 (2000).

10 Pacholczak, A., Zajaczkowska, M. & Nowakowska, K. The effect of brassinosteroids on rootting of stem cuttings in two barberry (*Berberis thunbergi*i L.) cultivars. *Agronomy-Basel* **11** (2021).

11 Keren, L. *et al.* Promoters maintain their relative activity levels under different growth conditions. *Mol. Syst. Biol.* **9**, 17 (2013).
