# Supplementary material for: Manipulation of sterol homeostasis for the production of 24-epi-ergosterol in industrial yeast
Source: Nat Commun. 2023 Jan 27;14:437. doi: 10.1038/s41467-023-36007-z (PMC9883489; doi:10.1038/s41467-023-36007-z)
Supplement: Supplementary file 3 — Description of Additional Supplementary Files [file 41467_2023_36007_MOESM3_ESM.pdf]

Description of Additional Supplementary Files

File name: Supplementary Data 1

Description: Heterologous gene sequences used in this study.

File name: Supplementary Data 2

Description: Strains used in this work.

File name: Supplementary Data 3

Description: Plasmids used in this work.

File name: Supplementary Data4

Description: Primers used in this work.
