# Supplementary material for: Manipulation of sterol homeostasis for the production of 24-epi-ergosterol in industrial yeast
Source: Nat Commun. 2023 Jan 27;14:437. doi: 10.1038/s41467-023-36007-z (PMC9883489; doi:10.1038/s41467-023-36007-z)
Supplement: Supplementary file 8 — Reporting Summary [file 41467_2023_36007_MOESM8_ESM.pdf]

nature portfolio

Corresponding author(s): Jianping Lin & Jiazhang Lian

Last updated by author(s): Jan 9, 2023

Reporting Summary

Nature Portfolio wishes to improve the reproducibility of the work that we publish. This form provides structure for consistency and transparency in reporting. For further information on Nature Portfolio policies, see our Editorial Policies and the Editorial Policy Checklist.

Statistics

For all statistical analyses, confirm that the following items are present in the figure legend, table legend, main text, or Methods section.

n/a	Confirmed	
☐	☒	The exact sample size (*n*) for each experimental group/condition, given as a discrete number and unit of measurement
☐	☒	A statement on whether measurements were taken from distinct samples or whether the same sample was measured repeatedly
☐	☒	The statistical test(s) used AND whether they are one- or two-sided<br>*Only common tests should be described solely by name; describe more complex techniques in the Methods section.*
☒	☐	A description of all covariates tested
☒	☐	A description of any assumptions or corrections, such as tests of normality and adjustment for multiple comparisons
☐	☒	A full description of the statistical parameters including central tendency (e.g. means) or other basic estimates (e.g. regression coefficient) AND variation (e.g. standard deviation) or associated estimates of uncertainty (e.g. confidence intervals)
☐	☒	For null hypothesis testing, the test statistic (e.g. *F*, *t*, *r*) with confidence intervals, effect sizes, degrees of freedom and *P* value noted<br>*Give P values as exact values whenever suitable.*
☒	☐	For Bayesian analysis, information on the choice of priors and Markov chain Monte Carlo settings
☒	☐	For hierarchical and complex designs, identification of the appropriate level for tests and full reporting of outcomes
☒	☐	Estimates of effect sizes (e.g. Cohen's *d*, Pearson's *r*), indicating how they were calculated

Our web collection on statistics for biologists contains articles on many of the points above.

Software and code

Policy information about availability of computer code

Data collection	Agilent 1100 Series HPCORE ChemStation was uesd to collect HPLC data; AB Sciex Triple TOF 5600+ analyst TF 1.6 was used to collect LC-TOF-MS/MS data; BRUKER DMX-500 TopSpin 2.1 was used to collect NMR data; Gene 9600 was used to collect qPCR data; WinCC Runtime loader was used to control 2 L fed-batch bioreactors and collect dissolved oxygen data.
Data analysis	Excel (Microsoft office 365) and Origin 2019b for quantitative analysis of data; Mestrelab.Mnova 14 was used to analyze NMR data; AlphaFold2 (https://colab.research.google.com/github/sokrypton/ColabFold/blob/main/AlphaFold2.ipynb#scrollTo=kOblAo-xetgx) was used to predict the structure of ArDWF1 and its mutants; PyMol v2.2.3.0 was used to analyze protein structures; Agilent 1100 Series HPCORE ChemStation was used to analyze HPLC data; AB Sciex PeakView® V2.2 was used to analyze LC-TOF-MS/MS data; E-CRISP (http://www.e-crisp.org/E-CRISP/) was used to design donors for the CRISPR/Cas9 system; Web BLAST in NCBI was used to analyze the PSI-BLAST of DWF1 sequences; Clustal Omega (https://www.ebi.ac.uk/Tools/msa/clustalo/) was used for Multiple Sequence Alignment.

For manuscripts utilizing custom algorithms or software that are central to the research but not yet described in published literature, software must be made available to editors and reviewers. We strongly encourage code deposition in a community repository (e.g. GitHub). See the Nature Portfolio guidelines for submitting code & software for further information.

Data

Policy information about availability of data

All manuscripts must include a data availability statement. This statement should provide the following information, where applicable:

- Accession codes, unique identifiers, or web links for publicly available datasets
- A description of any restrictions on data availability
- For clinical datasets or third party data, please ensure that the statement adheres to our policy

The authors declare that all data supporting the findings of this study are available in the manuscript and supporting information. All the protein structures are obtained from Alpha fold2. Source data are provided with this paper. Heterologous gene sequences, strains, plasmids and primers used in this study are provided in Supplementary Data 1-4, respectively.

Human research participants

Policy information about studies involving human research participants and Sex and Gender in Research.

Reporting on sex and gender	Not Applicable.
Population characteristics	Not Applicable.
Recruitment	Not Applicable.
Ethics oversight	Not Applicable.

Note that full information on the approval of the study protocol must also be provided in the manuscript.

Field-specific reporting

Please select the one below that is the best fit for your research. If you are not sure, read the appropriate sections before making your selection.

☒ Life sciences ☐ Behavioural & social sciences ☐ Ecological, evolutionary & environmental sciences

For a reference copy of the document with all sections, see nature.com/documents/nr-reporting-summary-flat.pdf

Life sciences study design

All studies must disclose on these points even when the disclosure is negative.

Sample size	By following the common standard within the field, for example, in Xu, Y. M. et al. De novo biosynthesis of rubusoside and rebaudiosides in engineered yeasts. Nature Communications 13, 3040 (2022), and in Shen, B. et al. Fermentative production of Vitamin E tocotrienols in Saccharomyces cerevisiae under cold-shock-triggered temperature control. Nature Communications 11, 5155(2020), which has also been indicated in the manuscript, the sample size of each set of data is biological triplicates (n=3). Specifically, three independent colonies of the same strain were tested under the same fermentation conditions, and the corresponding results were used to calculate the mean and standard deviation.
Data exclusions	All data were included for analysis in this study.
Replication	All values presented are the means of three biological replicates. All attempts at replication were successful.
Randomization	Experiments involving biological replicates started with colony picking from an agar plate randomly, and there is no control over which cells will be selected. Experimental replication is only sought to assess reproducibility and thus had no elements of randomization. In addition, experimental variables were changed on factor at a time (e.g. a specific gene is modified or not), while keeping all other factors identical. Therefore, randomization should be irrelevant to this study.
Blinding	No blinding was involved as it was not relevant to this study, because No animal or human research participants were utilized and all samples were processed in parallel. Blinding during group allocation is also irrelevant in this study because yeast colonies were randomly picked from an agar plate and there is no control over which cells will be selected and thus there is no bias during group allocation.

Reporting for specific materials, systems and methods

We require information from authors about some types of materials, experimental systems and methods used in many studies. Here, indicate whether each material, system or method listed is relevant to your study. If you are not sure if a list item applies to your research, read the appropriate section before selecting a response.

Materials & experimental systems

n/a	Involved in the study
☒	☐ Antibodies
☒	☐ Eukaryotic cell lines
☒	☐ Palaeontology and archaeology
☒	☐ Animals and other organisms
☒	☐ Clinical data
☒	☐ Dual use research of concern

Methods

n/a	Involved in the study
☒	☐ ChIP-seq
☒	☐ Flow cytometry
☒	☐ MRI-based neuroimaging
